# Effect of Different Diets on Lifetime of Brown-Banded Cockroaches, *Supella longipalpa* (Blattodea: Blattellidae)

**Published:** 2017-05-27

**Authors:** Hadis Mosayebian, Hamid Reza Basseri, Mojgan Baniardalani, Yavar Rassi, Hossein Ladonni

**Affiliations:** Department of Medical Entomology and Vector Control, School of Public Health, Tehran University of Medical Sciences, Tehran, Iran

**Keywords:** *Supella longipalpa*, Lifetime, Diet, Iran

## Abstract

**Background:**

The brown-banded cockroach, *Supella longipalpa*, is not as common as the German cockroach in Iran. This species seeks out areas that are very warm most of the time, and prefer warmer area than what German cockroaches prefer. There is relationship between development of instars and diet of cockroaches. The aim of this study was to determine the effect of different diets on biology, life cycle on nymphal stages of *S. longipalpa* in laboratory condition prior to investigate the insecticide resistance status of this species in residence area in Iran.

**Methods:**

The cockroaches were reared in the insectary of School of Public Health, Tehran University of Medical Sciences, condition and the population divided in four equal groups. The effect of four different diets on life cycle of *S. longipalpa* was studied to determine the effect of them on the lifetime of each nymphal stage.

**Results:**

The diets significantly affected on growth and development of immature life stages of *S. longipalpa*. Based on introduced diets to the cockroache populations, total immature life cycle was 54, 58, 60 and 66 d for diets 2,4,1, and 3 relatively. However, the overall lifetime of *S. longipalpa* in average was about 225 days.

**Conclusion:**

As far as urban pest control is concerned, the result of this study will facilitate any operational programs for control of *S. longipalpa*. Among the different tested diets, diets 2 and 4 with less duration would be recommended for rearing of *S. longipalpa* in laboratory condition.

## Introduction

About 3,500 species of cockroaches exist worldwide (World Health Organization 1999). During recent years brown-banded as a serious pest has been collected in different residential area in Tehran (personal communication, Department of Medical Entomology, School of Public Health, Tehran University of Medical Sciences).

The brown-banded cockroach is an important pest species with virtually little known of the interaction between feeding, diet composition, and reproduction and its insecticide resistance spectrum as well. Like German cockroach, the brown-banded cockroach is small and must feed prior to oothecal production. However, unlike German cockroach (*Blattella germanica*) females, which carry the ootheca for a protracted period, Brown- banded cockroaches (*Supella longipala*) females oviposit new ootheca every several days. Therefore, the effect of nutrient limitation should become evident much sooner in this species than in *B. germanica*. Brown-banded cockroaches build up their highest populations in high temperature areas. They do not need as much water as German cockroach, so they often live in locations, which are drier. They are often found in locations at eye-level or above (Hamilton et al. 1988). Mating in female German cockroach is inhibited during starvation (Roth and Stay 1962) and delayed when they are fed with a stressfully high protein diet (Hamilton and Schal 1988). In the absence of an adequate food supply, females either delay reproduction (Kunkel 1966, Durbin and Cochran 1985) or produce fewer and smaller ootheca (Mueller 1978).

Basic knowledge and understanding of the life cycle, behavior and habitat of Brown-banded cockroaches are essential, before conducting any operational programs for any pest control strategy in infested area. Though brown-banded cockroach is going to be more popular as the main urban pest, still there is poor evidence regarding to relationship between development of instars and diet of *S. longipalpa*.

The aim of this study was to find out the influence of different diets with carbohydrates based on some biological features such as survival and longevity of *S. longipalpa* in the laboratory condition.

## Materials and Methods

### Cockroach populations

Brown banded cockroaches were collected from an infested area in Tehran, Iran. All cockroaches were maintained in an insectary of School of Public Health, Tehran University of Medical Sciences at 27±2 °C, 60±10 % RH, with a photoperiod of 12: 12h (L: D). Each population of cockroach was reared in the same size labeled glass jar. The cockroaches were provided with four different diets and water.

### Experimental design and diets

In order to avoid any heterogeneity in population, the trial was conducted on 2^nd^ generation of Brown-banded Cockroaches. Newly emerged unfed nymphs (aged 3+5 days) of cockroaches were randomly allocated to each type of diets. The effect of each type of food staffs e.g. milk, date, and both (date+milk) on longevity of immature stage of cockroaches were studied in the present of normal diet (dry bread and dry pellets of rabbit food, bran and water), i.e. the diet, regularly was used for rearing and maintaining the cockroach colonies in our laboratory. The diets used in this study were: 1) normal diet, 2) diet 1 + date, 3) diet1 + milk, and 4) diet 1 + date and milk. Each group of insects consisted of 10 cockroaches placed in a plastic buckle (3 liters capacity) and then the insects were allowed to feed based on the selected diets (as above) available in a plastic Petri dish. Each test was consisted of three replications.

### Statistical analysis

Kaplan-Meier statistical test (particular statistical test, recommended for survival test in biology) and one way analyzes of variance (ANOVA) were used to find out how the effect of different diets on life time of nymphal stages of *S. longipalpa* varies, using SPSS statistical package 18 (Chicago, IL, USA). The earlier statistical methods have been selected based on experimental design, diets, and variables applied in this study.

## Results

### Effect of diets on nymphal stages

The diets significantly affected on growth and development of immature life stages of *S. longipalpa* (Kaplan-Meier statistical test, P< 0.05). Based on introduced diets to the cockroach populations, total immature life cycle was 54, 58, 60 and 66 d for diets 2,4,1, and 3, respectively. *S. longipalpa* had 4 distinct nymphal stages for immature stage prior to adult stag, i.e. overall, 5 stages including adult. The total nymphal stages were completed within 59.1 d±0.32 (days ±S.E) for diet 1. The result for each nymphal stage 1, 2, 3 and 4 stages was 13.2±0.18, 15.03±0.37, 18.87±0.46 and 12±0.27days, respectively. The group of cockroaches, which used diet 2, completed all nymphal stages within 54.07±0.27 days. In addition, duration of each nymphal stage 1, 2, 3 and 4 were 12.77±0.16, 13.13±0.24, 17.77±0.39 and 10.40±0.25 days respectively. The insects which fed on diet 3, completed the total nymphal stages within 66.43±0.45 days and duration of nymphs for each stage of 1, 2, 3 and 4 was 14.03±0.15, 15.63±0.28, 21.40±0.84 and 15.37±0.55 respectively. The cockroaches which diet 4 was provided for them, completed whole nymphal stage within 57.99±0.3 days. The duration of development for each stage of 1, 2, 3 and 4 was 13.03±0.18, 14.7±0.26, 18.53±0.51 and 11.73±0.27 respectively (Table 1).

**Table 1.** The effect of different diets on lifetime of nymphal stages of *Supella longipalpa* in laboratory (Kaplan meier test)

**Diets**	**D1**	**D2**	**D3**	**D4**

**Days**				
**N1**	13.20±0.18	12.77±O.16	14.30±0.15	13.03±0.18
	1*	1*	1*	1*
**N2**	15.03±0.37	13.13±0.24	15.63±0.28	14.70±0.26
	2*	2*	2*	2*
**N3**	18.87±0.46	17.77±0.39	21.40±0.84	18.53±0.51
	3*	3*	3*	3*
**N4**	12±0.27	10.40±0.25	15.37±0.55	11.73±0.27
	4*	4*	4*	4*

N 1, N2, N3, N4 represent 1^st^, 2^nd^, 3^rd^ and 4^th^ nymphal stage of *Supella longipalpa.*

D1, D2, D3and D4 represent the type of diets, D1: Normal diet (dry bread-dry pellets of rabbit food, bran and water), D2: diet 1 + date. D3: diet 1+ milk, D4: diet 1+ (date and milk) and respectively (P< 0.05). The numbers represent the mean value days of development of nymphal stage from three replicates where the standard errors are included. The number of insects in each group of replication was 10 cockroaches (n= 10).

(*): Represent the significant differences between Nymphal stages and different diets using Kaplan Meier statistical tes t< 0.05.

**Fig. 1.**
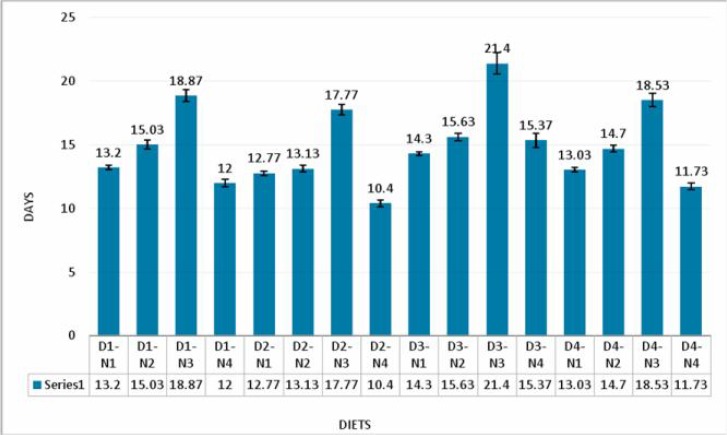
The effect of different diets on lifetime of nymphal stages of *Supella longipalpa* in laboratory (ANOVA test). The bars represent the mean value days of development of nymphal stage from three replicates where the standard errors are included. N 1, N2, N3, N4 represent 1^st^, 2^nd^, 3^rd^ and 4^th^ nymphal stage *Supella longipalpa* (F.) and D1, D2, D3and D4 represent the type of diets provided for each group of cockroaches D1: Normal diet (dry bread-dry pellets of rabbit food, bran and water), D2: diet 1+ date, D3: diet 1+milk, D4: diet 1+ (date and milk) respectively.

Generally, the shortest stage occurred among forth instar stage, which the longest belonged to third instars (Fig. 1). To compare the duration of first instar development, those insects, which fed on diet 2, had shortest lifetime while diet 3 caused longest durations among four groups. The maximum stage occurred in third instar nymph, which fed with diet 3 (Fig. 1).

**Fig. 2.**
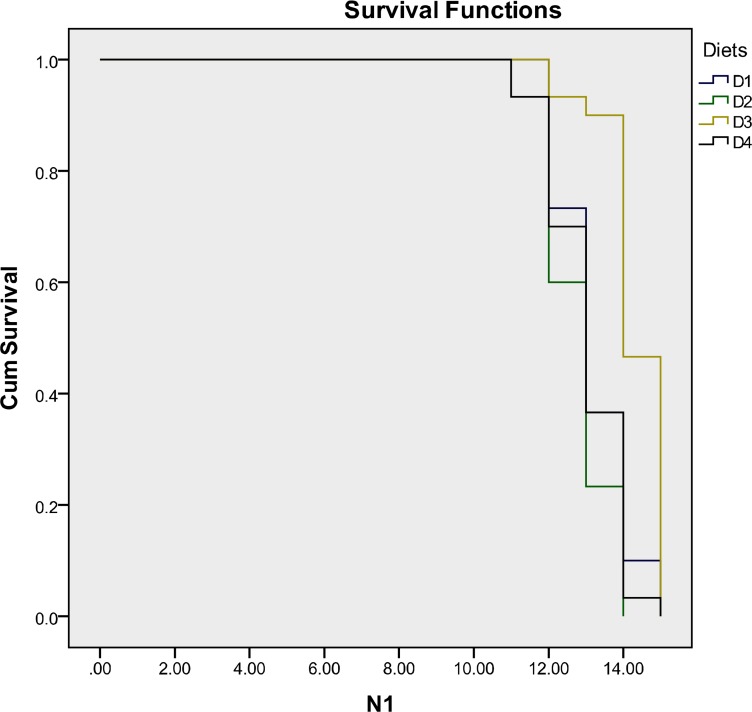
Effect of different diets on 1^st^ nymphal stage of *Supella longipalpa* (Kaplan Meier, survival test) N1: 1^st^ nymphal stage D1, D2, D3 and D4 represent the type of diets, D1: Normal diet (dry bread-dry pellets of rabbit food, bran and water), D2: diet 1 + date, D3: diet 1+milk, D4: diet 1 + date and milk and the nypmphal stages respectively. The number of insects in each group of replication was 10 cockroaches (n= 10)

**Fig. 3.**
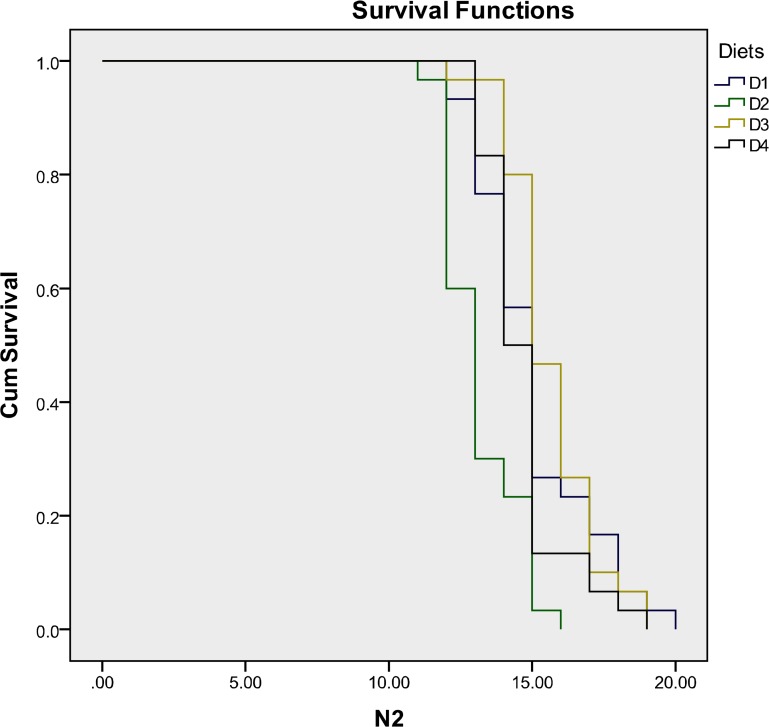
Effect of different diets on 2^nd^ nymphal stage of *Supella longipalpa* (Kaplan Meier, survival test) N2: 2^nd^ nymphal stage. D1, D2, D3and D4 represent the type of diets, D1: Normal diet (dry bread-dry pellets of rabbit food, bran and water), D2: diet 1 + date, D3: diet 1 + milk, D4: diet 1+date and milk and the nypmphal stages respectively. The number of insects in each group of replication was 10 cockroaches (n= 10)

**Fig. 4.**
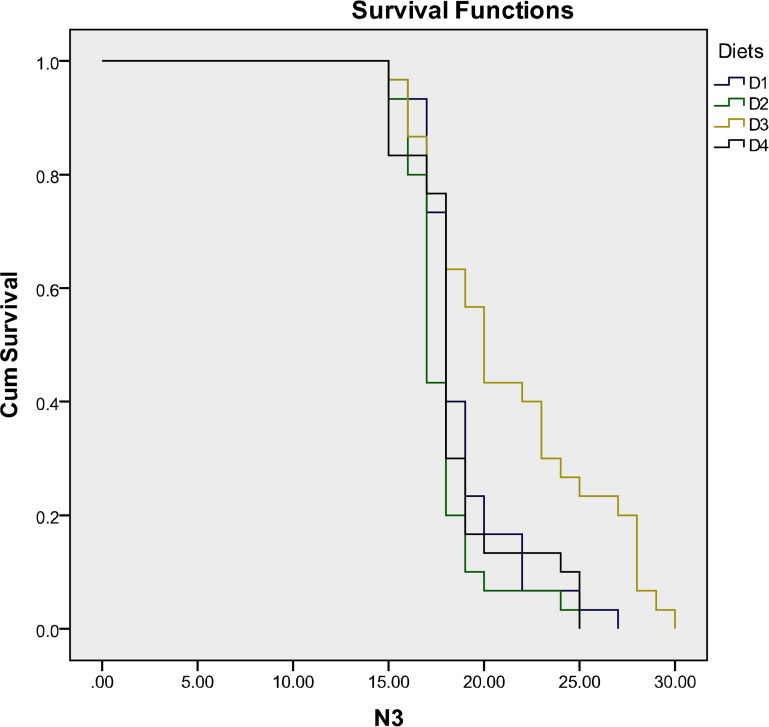
Effect of different diets on 3^rd^ nymphal stage of *Supella longipalpa* (kaplan Meier, survival test). D1, D2, D3and D4 represent the type of diets, D1: Normal diet (dry bread-dry pellets of rabbit food, bran and water), D2: diet 1 + date, D3: diet 1+ milk, D4: diet 1 + date and milk and the nypmphal stages respectively. The number of insects in each group of replication was 10 cockroaches (n = 10) N3: 3^rd^ nymphal stage.

**Fig. 5.**
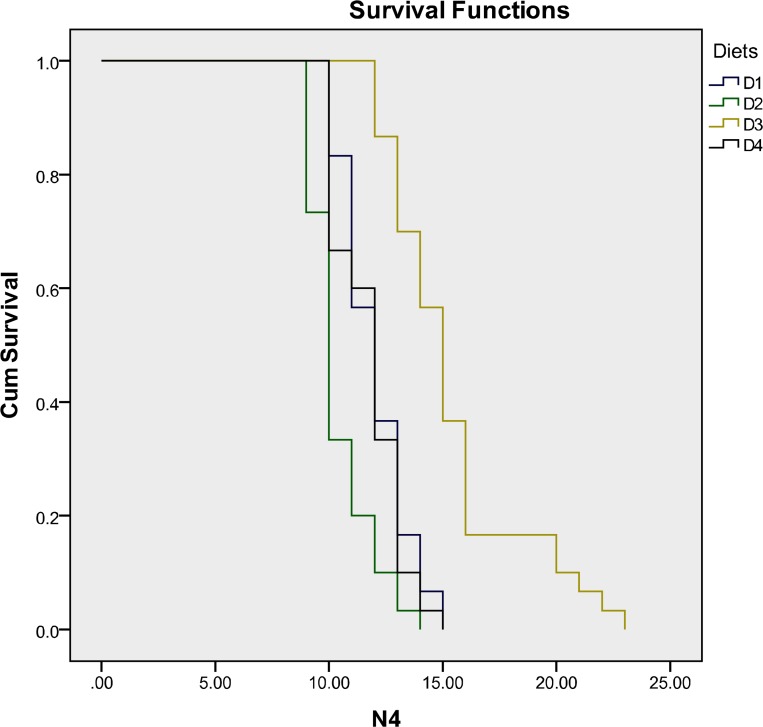
Effect of different diets on 4^th^ nymphal stage of *Supella longipalpa* (Kaplan Meier, survival test) D1, D2, D3 and D4 represent the type of diets, D1: Normal diet (dry bread-dry pellets of rabbit food, bran and water), D2: diet 1 + date, D3: diet 1 + milk, D4: diet 1 + date and milk and the nypmphal stages respectively. The number of insects in each group of replication was 10 cockroaches (n = 10). N4: 4^th^: nymphal stage

The cumulated survival of nymphs moulted from each stage to next based on diet per time is presented in Fig. 2–5. The number of nymphs moved to next stage was varied within stages and between four diets. Although the fist instar of insect group, which fed on diet 1 started moulting earlier, the group, which had diet 2, completed first instar development in a shorter time. In addition, diet 3 cause delays in the moulting of 1^st^ instar of cockroach (Fig. 2).

Diet 2 accelerated movement of 2^nd^ instar to next stage faster comparing with others. The duration rage for completing 2^nd^ instar in this group occurred between 11 to 16 days while the insects used diet 1 stated moulting from 13^th^ and finished on 20^th^ (Fig. 3). Generally, duration of 3^rd^ instar was longer than other stages in four groups. Although four types of the diet accelerated moulting of 3^rd^ instar to next stage within nearly same time, the diet 3 showed delay in development of this stage longer than other groups and the last cockroaches moved to 4^th^ instar at day 30^th^ (Fig. 4). The duration 4^th^ instar development also showed variation while the diet 2 started to move to adult stage faster and the number of insect, which completed this stage was shorter with diet 2 as well. In contrary, the cockroaches treated with diet 3 started moulting later and the duration of 4^th^ instar was obviously longer comparing with other groups (Fig. 5).

## Discussion

We found that modification of diet can significantly effect on duration of nymphal development of *S. longipalpa*. Diet is one of the most crucial factor to rear or colonizing insects in laboratory condition. Generally, feeding insect with appropriate diet can reduce the duration of instar development to minimum time and optimize the reproductions.

Four diets were introduced to immature stages of *S. longipalpa* with types of diets based on the longevity of nymphal stages. The shorter life time/day indicating the appropriated diet was given to the insects.

Colonies of cockroach facilitate field of researches on pest control such as bioassay tests and evaluation of insecticides. In terms of cockroach and an emphasis on IVM control, comprehensive studies have been carried out on different aspects of German cockroach *B. germanica* (L.) (Cochroan 1986, Ladonni et al. 1988, Ladonni 2000, Ladonni 2001). Inheritance and resistance spectrum, molecular basis of kdr resistance of German cockroach to four main groups of insecticides have been widely studied on different Iranian strains of German cockroach (Ladonni et al. 1988, Limoee et al. 2007, Limoee et al. 2011). There are some evidences indicating that *S. longipalpa* has been substitution with German cockroach (*Blattella germanica*) in some cities of Iran. Therefore, *S. longipalpa* is going to be more considered as an urban pest in many cities while poor knowledge are available about biology and ecology if this species.

Studies with German cockroach have revealed an intimate association between the availability of food and water and reproductive success (Roth and stay 1962, Kunkel 1966, Mueller 1978). Food and water consumption are cyclical and closely related to the reproduction cycle in German and American cockroaches (Bell 1969, Cochran 1983, Rollo 1984, Durbin and Cochran 1985, Hamilton and Schal 1988). However, little studies have been carried out on resting and feeding behavior of *S. longipalpa*.

Influence of dietary protein on food intake and reproduction of female German cockroach was studied by Hamilton and Schal (1988), so adult performance was directly influenced by deity protein level. Females which had a diet with 65% protein, died rapidly while the females fed on nutrient which contained 5% protein, not only their reproductive rate reduced but also the size of oothecaes were smaller.

Our results indicated that food staffs based on hydrocarbon compounds has a great effect on development of immature stage. The results of cumulated survival analyses on nymphs’ development indicated that passing nymphes from one stage to next was varied based on the type of diet. This variation could be due to carbohydrate food available particularly those insects, which accessed to date. It seems that date as supplementary food could accelerate the nymphs of cockroaches to complete each stage faster. Date contain high amount simple carbohydrates that insects can quickly digests and turns into energy. Therefore, this study recommends date as supplementary food for feeding cockroaches particularly for rearing *S. longipalpa* in laboratory condition.

## Conclusion

As far as host pest control is concerned, colonizing pest at optimum condition is essential for pesticides evaluating and understanding of other aspects of biology and ecology of pest such as *S. longipalpa.* Accordingly, the optimum diets for accelerating moulting of brown-banded instars are diets 2 and 4.
